# Regulation of self-renewal and senescence in primitive mesenchymal stem cells by Wnt and TGFβ signaling

**DOI:** 10.1186/s13287-023-03533-y

**Published:** 2023-10-26

**Authors:** Matteo Mazzella, Keegan Walker, Christina Cormier, Michael Kapanowski, Albi Ishmakej, Azeem Saifee, Yashvardhan Govind, G. Rasul Chaudhry

**Affiliations:** 1 Department of Biological Sciences, Oakland University, Rochester, MI 48309 USA; 2 OU-WB Institute for Stem Cell and Regenerative Medicine, Rochester, MI 48309 USA

**Keywords:** Mesenchymal stem cells, Self-renewal, Proliferation, Differentiation, Senescence, Wnt pathway, TGFβ signaling

## Abstract

**Background:**

The therapeutic application of multipotent mesenchymal stem cells (MSCs) encounters significant challenges, primarily stemming from their inadequate growth and limited self-renewal capabilities. Additionally, as MSCs are propagated, their ability to self-renew declines, and the exact cellular and molecular changes responsible for this are poorly understood. This study aims to uncover the complex molecular mechanisms that govern the self-renewal of primitive (p) MSCs.

**Methods:**

We grew pMSCs using two types of medium, fetal bovine serum (FM) and xeno-free (XM), at both low passage (LP, P3) and high passage (HP, P20). To evaluate LP and HP pMSCs, we examined their physical characteristics, cell surface markers, growth rate, colony-forming ability, BrdU assays for proliferation, telomerase activity, and potential to differentiate into three lineages. Moreover, we conducted RNA-seq to analyze their transcriptome and MNase-seq analysis to investigate nucleosome occupancies.

**Results:**

When grown in FM, pMSCs underwent changes in their cellular morphology, becoming larger and elongated. This was accompanied by a decrease in the expression of CD90 and CD49f, as well as a reduction in CFE, proliferation rate, and telomerase activity. In addition, these cells showed an increased tendency to differentiate into the adipogenic lineage. However, when grown in XM, pMSCs maintained their self-renewal capacity and ability to differentiate into multiple lineages while preserving their fibroblastoid morphology. Transcriptomic analysis showed an upregulation of genes associated with self-renewal, cell cycle regulation, and DNA replication in XM-cultured pMSCs, while senescence-related genes were upregulated in FM-cultured cells. Further analysis demonstrated differential nucleosomal occupancies in self-renewal and senescence-related genes for pMSCs grown in XM and FM, respectively. These findings were confirmed by qRT-PCR analysis, which revealed alterations in the expression of genes related to self-renewal, cell cycle regulation, DNA replication, differentiation, and senescence. To understand the underlying mechanisms, we investigated the involvement of Wnt and TGFβ signaling pathways by modulating them with agonists and antagonists. This experimental manipulation led to the upregulation and downregulation of self-renewal genes in pMSCs, providing further insights into the signaling pathways governing the self-renewal and senescence of pMSCs.

**Conclusion:**

Our study shows that the self-renewal potential of pMSCs is associated with the Wnt pathway, while senescence is linked to TGFβ.

**Supplementary Information:**

The online version contains supplementary material available at 10.1186/s13287-023-03533-y.

## Background

Mesenchymal stem cells (MSCs) are versatile stem cells that can be extracted from various adult and perinatal sources. The Mesenchymal and Tissue Stem Cell Committee of the International Society for Cellular Therapy has established criteria to define these cells [1, 2]. MSCs typically have a fibroblastoid appearance, adhere to surfaces, and express a particular cluster of differentiation (CD) markers, such as CD73, CD90, and CD105. They possess the remarkable ability to differentiate into chondrogenic, osteogenic, and adipogenic lineages [3–5]. Recent studies have expanded their potential to differentiate into neural stem cells, retinal progenitor cells, and myocytes [6–8].

MSCs, aside from their ability to differentiate, have therapeutic benefits. They possess anti-inflammatory and immunomodulatory properties that enable them to travel to areas of injury and provide beneficial effects [9]. A significant advantage of MSCs is that they offer a viable alternative to ESCs, which are mired in ethical issues and can potentially develop teratomas [10, 11]. However, there are challenges associated with utilizing MSCs for cell therapy. MSCs exhibit suboptimal growth characteristics, gradually lose their self-renewal capacity, and undergo senescence with successive passaging. These factors hold significant implications for the sustainability, efficacy, safety, and long-term benefits of MSC-based regenerative medicine.

In addition, culture media that frequently use fetal bovine serum (FBS) can bring about alterations in the morphology and self-renewal abilities of MSCs, as well as other characteristics such as their immunomodulatory functions [12, 13]. The composition of FBS is inconsistent due to various extraction methods and may have unknown xenogeneic pollutants [14, 15]. Additionally, animal sera can be infected by viruses, mycoplasma, prions, and endotoxins [15, 16], leading to concerns about their usage in culture media.

In response to these challenges, xeno-free medium (XM) has emerged as a promising alternative for MSC culture [17–21]. XM comprises human serum and proteins, offering potential advantages in maintaining MSC self-renewal [22–24].

Maintaining MSCs during propagation is complicated due to a lack of understanding about the molecular changes that affect their ability to self-renew and differentiate after passaging or culturing. This knowledge gap has caused variations in preclinical and clinical studies that use MSCs, due to differences in cell source, media composition, and culture conditions [4, 25]. Although it is known that passaging causes senescence in MSCs [26, 27], the specific mechanisms are still unclear.

Previous studies have indicated that Wnt signaling is vital for stem cell self-renewal and proliferation [3, 4, 28]. Whereas TGFβ signaling regulates a range of cellular processes, such as proliferation and differentiation [29], Wnt signaling is a crucial pathway that maintains stem cell properties by inhibiting differentiation, regulating the cell cycle, and maintaining the stem cell niche [30, 31]. However, the TGF-β signaling can cause senescence in MSCs through a range of mechanisms such as cell cycle arrest, activation of senescence-associated markers, SASP secretion, activation of the p53 pathway, and DNA damage response [32, 33]. Understanding molecular changes and associated mechanisms may lead to better maintenance of MSC self-renewal and more reproducible results in MSC-based research and therapies. It is also crucial to comprehend these mechanisms to utilize the regenerative capabilities of MSCs in therapeutic applications like tissue engineering and regenerative medicine.

Our study focused on investigating the intricate molecular mechanisms that regulate the self-renewal process of primitive (p) MSCs obtained from the human umbilical cord [4]. These pMSCs possess a high capacity for cell division, are naive, and have a low risk of triggering an immune response in the host due to their lack of major histocompatibility complex class II antigen expression [6]. Unlike adult MSCs, umbilical cord-derived MSCs typically do not cause graft vs. host disease [3, 6].

Through our research, we discovered that the upregulation of Wnt signaling, cell cycle-related genes, and genes involved in DNA replication play a critical role in maintaining the self-renewal and differentiation potential of pMSCs. On the other hand, the upregulation of TGFβ signaling leads to senescence in pMSCs. These findings have exciting implications for expanding pMSC populations while preserving their properties, which could have significant therapeutic and pharmacological applications.

## Materials and methods

### Culturing of pMSCs

For this study, we utilized umbilical cord MSCs that were well-characterized and previously described [5]. The umbilical cord tissue was obtained from healthy donors who had given their consent through the Ascension Providence Hospital in Southfield, MI under an approved IAA protocol (IAA #400244-10). These cells were maintained by passaging in FM (DMEM nutrient mix F12 medium, Life Technologies, Carlsbad, CA, USA) supplemented with 10% fetal bovine serum (VWR, Radnor, PA, USA) and 5.6% antibiotic solution consisting of 0.1% gentamicin, 0.2% streptomycin, and 0.12% penicillin (Sigma, St Louis, MO, USA) and XM (MSC NutriStem® XM, Sartorius, Goettingen, Germany) supplemented with MSC NutriStem® XF Supplement Mix (Sartorius, Goettingen, Germany) and 5.6% of the antibiotic solution. XM contained human serum, proteins, peptides, etc., but no animal products based on the provider's specifications. The pMSCs were considered low passage (LP) cells if they were grown for three passages (P) and high passage (HP) cells if they were passaged to P20. LP and HP pMSCs were cultured at a density of 1 × 10^4^/cm^2^ in FM and XM, respectively, until they reached 70% confluency in a CO_2_ incubator at 37 °C.

### Immunophenotyping

To analyze the cell surface markers, Flow cytometry was used for Immunophenotyping. The LP and HP pMSCs were cultured in their respective medium until they reached 70% confluency. After that, they were trypsinized and pelleted for analysis. FITC-conjugated antibodies against CD44, CD49f, and CD90 or APC-conjugated antibodies against CD29, CD73, and CD105 were used to label the cells singularly or dually. The stained cells were then analyzed on a FACS Canto II (Becton Dickinson) using Diva Software (Beckton Dickinson).

### Colony forming efficiency (CFE) assay

LP and HP pMSCs were cultured in FM and XM at a concentration of 100 cells in a 100 mm petri dish. After 10–14 days, the cells were washed with PBS and fixed in paraformaldehyde for 10 min. The fixed cells were stained with 0.1% crystal violet (Thermo Scientific) for 1 h and rinsed with tap water. Colonies were identified as groups of at least 50 cells. The results were recorded as the total number of colonies per number of plated cells in triplicate.

### Cellular proliferation assay

We suspended LP and HP pMSCs in FM and XM solutions and plated them at a concentration of 6.25 × 10^3^ cells per well in a 96-well plate. We left them to grow for 2 days. To measure their proliferation, we followed the manufacturer's guidelines and used the BrdU proliferation kit in triplicate (Novus Biologicals, Centennial, CO, USA). The BrdU assay is a commonly used method to evaluate changes in cell proliferation, as it can detect newly synthesized DNA.

### Telomerase assay

To determine telomerase activity, we used the telomere repeat amplification protocol (TRAP) assay [34–36]. First, we prepared whole cell lysates from cultured cell pellets and lysed them in cold RIPA buffer (Santa Cruz) with protease inhibitor cocktail and sodium orthovanadate at a ratio of 100 μL of buffer per 1.0 × 10^6^ cells. We cleared the lysates by centrifugation at 14,000 rpm for 10 min at 4 °C and stored them at − 80 °C. We determined the protein concentration using nanodrop 8000 (Thermofisher).

We used the SYBR green master mix (Promega) for the quantitative reverse transcriptase-polymerase chain reaction (qRT-PCR). In each well, we added 0.1 μg protein lysate, 50% volume SsoAdvanced SYBR green master mix (Bio-Rad), 0.1 μg of each primer TS (5′-AATCCGTCGAGCAGAGTT-3′), and ACX (5′-GCGCGG(CTTACC)3CTAACC-3′) (Integrated DNA Technologies), and RNase/DNase-free water to achieve a final well volume of 25 μL. We performed qRT-PCR and detection on a CFX 90 (Bio-Rad). In addition to the treatment samples, we included a series of controls on each plate: (1) no template control with TS primer only, (2) no template control with ACX primer only, (3) no template control with TS and ACX primers (used in the normalization of samples), (4) heat-inactivated control with the template (protein lysate) and TS and ACX primers, and (5) HEK cell lysate with TS and ACX primers (a positive control).

### Beta-galactosidase assay

The pMSCs were cultivated in a 12-well plate with a seeding density of 5.0 × 10^4^ per well for 48 h. To determine the senescence-associated beta-galactosidase (SA-β-gal) activity, a SA-β-gal staining kit (Cell Signaling Technology, Danvers, MA, USA) was used in accordance with the manufacturer's instructions. The stained cells were then examined using light microscopy.

### Trilineage differentiation and characterization

The LP and HP pMSCs were grown in FM and XM. After 24 h of plating the pMSCs, they were induced to differentiate using specific differentiation media for 3 weeks. The adipogenic differentiation medium was made up of DMEM nutrient mix F12, which contained 0.5 μM isobutyl-methylxanthine, 1 μM dexamethasone, 10 μM insulin, and 200 μM indomethacin. The chondrogenic differentiation medium consisted of DMEM nutrient mix F12, which contained 20 ng TGFβ1, 10 ng insulin, 100 nM dexamethasone, and 100 μM ascorbic acid. The osteogenic differentiation was made up of DMEM nutrient mix F12, which contained 0.1 μM dexamethasone, 10 μM β-glycerophosphate, and 50 μM ascorbate-phosphate. Control pMSCs were grown using FM or XM.

The differentiated cells were characterized using oil red o, toluidine blue, and alizarin red to identify adipogenic, chondrogenic, and osteogenic differentiation, respectively, as previously reported [4]. To determine the expression of specific proteins in the differentiated derivatives of pMSCs, they were fixed on coverslips by treating them with 4% paraformaldehyde for 10 min at room temperature. The fixed cells were then permeabilized with 0.5% Triton X-100 (Sigma), blocked in 2% bovine serum albumin (Sigma) in PBS for 1 h, and subjected to specific primary antibodies at 1:100 dilutions at 4 °C overnight. They were then treated with a secondary antibody at 1:200 dilution for 2 h at room temperature, counterstained with DAPI at 1:200 dilutions for 30 min at room temperature, and mounted onto a slide. Fluorescent images were captured using a confocal microscope (NIKON Instruments Inc.). The images were then quantified using ImageJ. To quantify the fluorescent intensity, the following equation was used: fluorescent intensity = integrated density − (area of selected cell x mean fluorescence of background readings).

### Treatment of pMSCs with Wnt and TGFβ agonist and antagonist

To study how Wnt agonist and antagonist affect pMSCs, we cultured the cells in FM and XM for 24 h before treating them with either 10 mM of Wnt agonist lithium chloride (LiCl) from Sigma or 500 ng of Wnt antagonist Fz7-21 TFA from MedChemExpress in Monmouth Junction, NJ, USA. The cells were then cultured for an additional 24 h before being analyzed.

We conducted a similar experiment to investigate the effects of TGFβ agonist and antagonist. pMSCs were plated for 24 h and then treated with either 0.25 mM of the TGFβ agonist BMP signaling agonist sb4 (BMPSB4) from MedChemExpress in Monmouth Junction, or 0.125 mM of the TGFβ antagonist Asiaticoside from the same supplier. The cells were then cultured for an additional 24 h before being analyzed..

### RNA-sequencing (RNA-seq)

To analyze the RNA from LP and HP pMSCs grown in FM and XM, we followed a published protocol [11]. The RNA was isolated, quantified, and qualified using Agilent2100 Bioanalyzer and Qubit Assay. RNA samples with a RNA integrity number (RIN) of 10 were used. We used the KAPA RNA HyperPrep Kit with RiboErase to prepare cDNA libraries per the manufacturer's instructions. The libraries were sent for RNA-seq transcriptome analysis by GENEWIZ (South Plainfield, NJ, USA), which performed 2 × 150 bp paired-end read sequencing on the Illumina NovaSeq/HiSeq. Each sample yielded an average of 47 million reads. The fragments were then mapped to the reference human genome assembly hg38, and differential gene expression analysis was performed using the Galaxy platform (https://usegalaxy.org/) [37]. We performed RNA-Seq analyses on three independent biological replicates.

### Micrococcal nuclease sequencing (MNase-seq)

To prepare the DNA libraries for analysis, formaldehyde was used to cross-link nucleosomes, which were then lysed. MNase digestion was employed to fragment the DNA, and the Illumina NEBNext Ultra II Library prep kit was used to process it. After gel purification, the libraries were sequenced at the University of Michigan Advanced Genomics Core Facility in Ann Arbor, MI, USA, using the Illumina HiSeq-4K in paired-end mode. To analyze the data, the paired-end reads were mapped to the human genome assembly hg38 and analyzed using the Galaxy platform. Three independent biological replicates were used for MNase-seq analysis.

### Bioinformatic analysis

To evaluate the quality of the raw reads in RNA-seq, we used the FastQC toolkit. Next, Trim Galore! was employed to trim low-quality reads and adapters. The raw reads were then mapped to the hg38 human genome using HiSat2. FeatureCounts was used to determine the expression of each gene. To normalize the raw counts, DESeq2 was utilized, and the expression was determined for the differential genes. Genes with an adjusted *p*-value of < 0.05 and a 1.5 fold for log2(FC) were deemed significant. To analyze the differential genes, we used protein analysis through evolutionary relationships (PANTHER) and the Kyoto Encyclopedia of Genes and Genomes (KEGG) pathway. Heatmapper (http://www.heatmapper.ca/) was used to generate heatmaps to assess expression.

To perform MNase-seq, we first isolated the adapter sequences from the genomic inserts present at the 5’ to 3’ ends of the reads. We then analyzed these sequences using Trim Galore! and mapped the isolated regions to the hg38 human genome with the help of Bowtie2. After alignment, we merged the Bowtie2 outputs using Merge SAM/BAM files. To compute the Scale Factor, we added the reads for each condition and divided the sum by the highest number of reads. The data was normalized using bamCoverage. To analyze transcripts, we used the UCSC Main Genome Browser. Next, we imported the files into the ComputeMatrix tool to create a matrix file. Finally, we visualized the data using plotHeatmap and Interactive Genomics Viewer (IGV).

### qRT-PCR

LP and HP pMSCs grown in FM and XM were harvested and subjected to the isolation of their total cellular mRNA using the GeneJET RNA purification Kit (Thermo Scientific), following the manufacturer’s instructions. The RNA was purified using a thermocycler (Bio-Rad), and the cDNA was synthesized using the iScript kit (Bio-Rad, Hercules, CA, USA). qRT-PCR was performed using SsoAdvanced SYBR Green Supermix (Bio-Rad) and the CFX90 Real-Time PCR system. The primers (IDT Technologies, Coralville, IA, USA) used for trilineage differentiation, self-renewal, cell cycle, DNA replication, and senescence in this study are in Additional file 1: Fig. S1. We conducted the reactions in triplicate and normalized them using the reference genes GAPDH and β-ACTIN.

### Statistical analysis

The data are expressed as mean ± standard error of the mean (SEM) of triplicates per analysis. Any results showing **p ≤ 0.01 and *p ≤ 0.05 were deemed statistically significant. All analyses were conducted using the one-way ANOVA test on SPSS version 26 (SPSS Inc. USA).

## Results

### Characteristics pMSCs grown in FM and XM

**Fig. 1:**
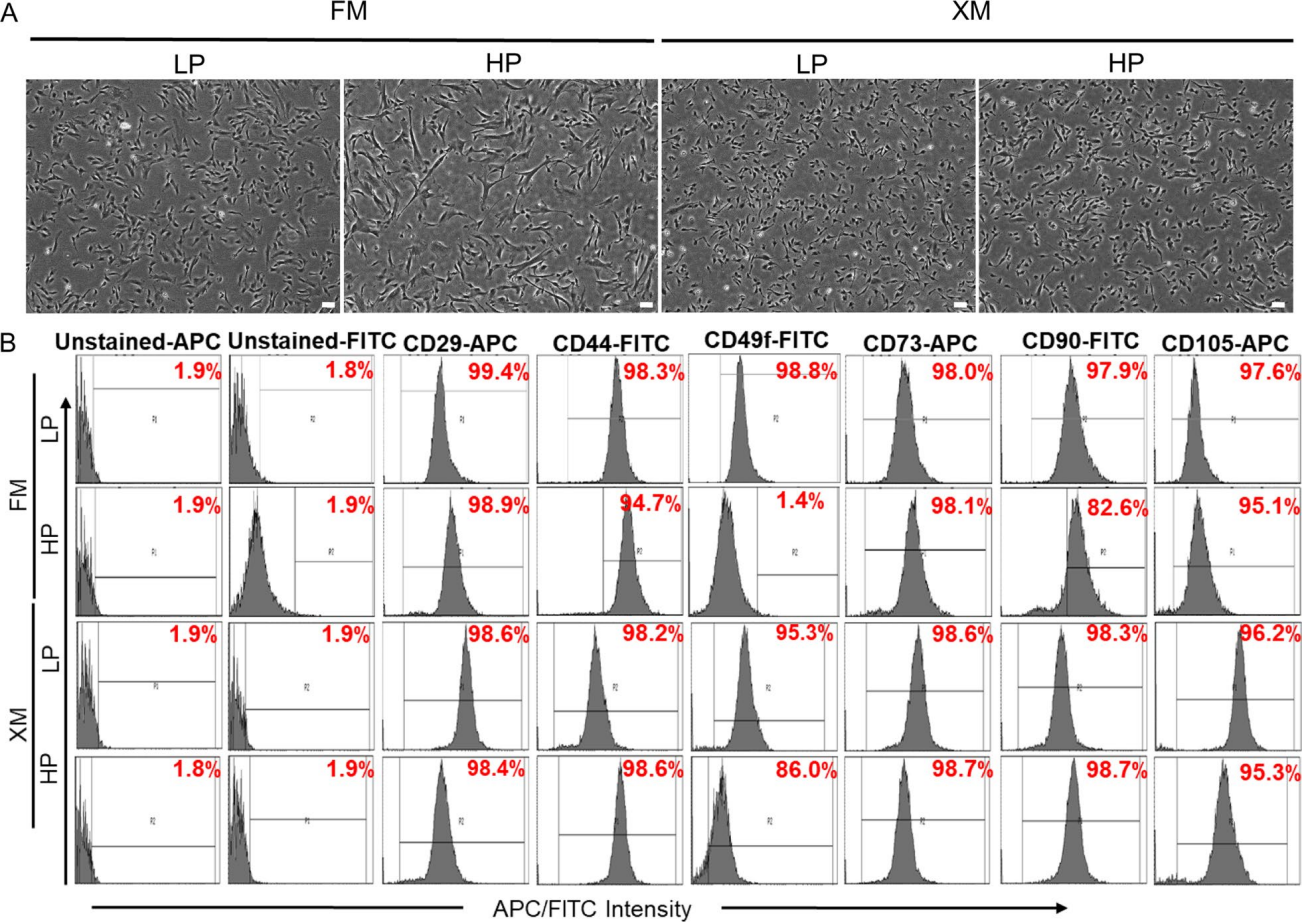
Characteristics of LP and HP pMSCs grown in FM and XM. **a**, **b** Phase contrast microscopy images and expression of surface markers determined by flow cytometer in pMSCs, respectively. Scale bars represent 100 μm (magnification: 4×). HP pMSCs in FM had larger cell size and significantly downregulated expression of CD49f and CD90 compared to LP pMSCs

Many reports indicate that MSCs progressively lose self-renewal and differentiation potential upon passaging [26, 27]. Our results showed that when pMSCs were repeatedly passaged using FM, they became increasingly larger and elongated morphology (Fig. 1a). In contrast, the XM maintained smaller fibroblastoid morphology even after twenty passages. FACS analysis of the LP and HP pMSCs showed maintenance of MSC-specific surface makers, CD29, CD44, CD49f, CD73, CD90, and CD105 in LP and HP pMSCs cultured in XM. While the expression of these markers in LP MSCs cultured in FM and XM was similar, the expression of CD49f and CD90 was significantly reduced in HP MSCs grown in FM (Fig. 1b and Additional file 2: Fig. S2).

**Fig. 2:**
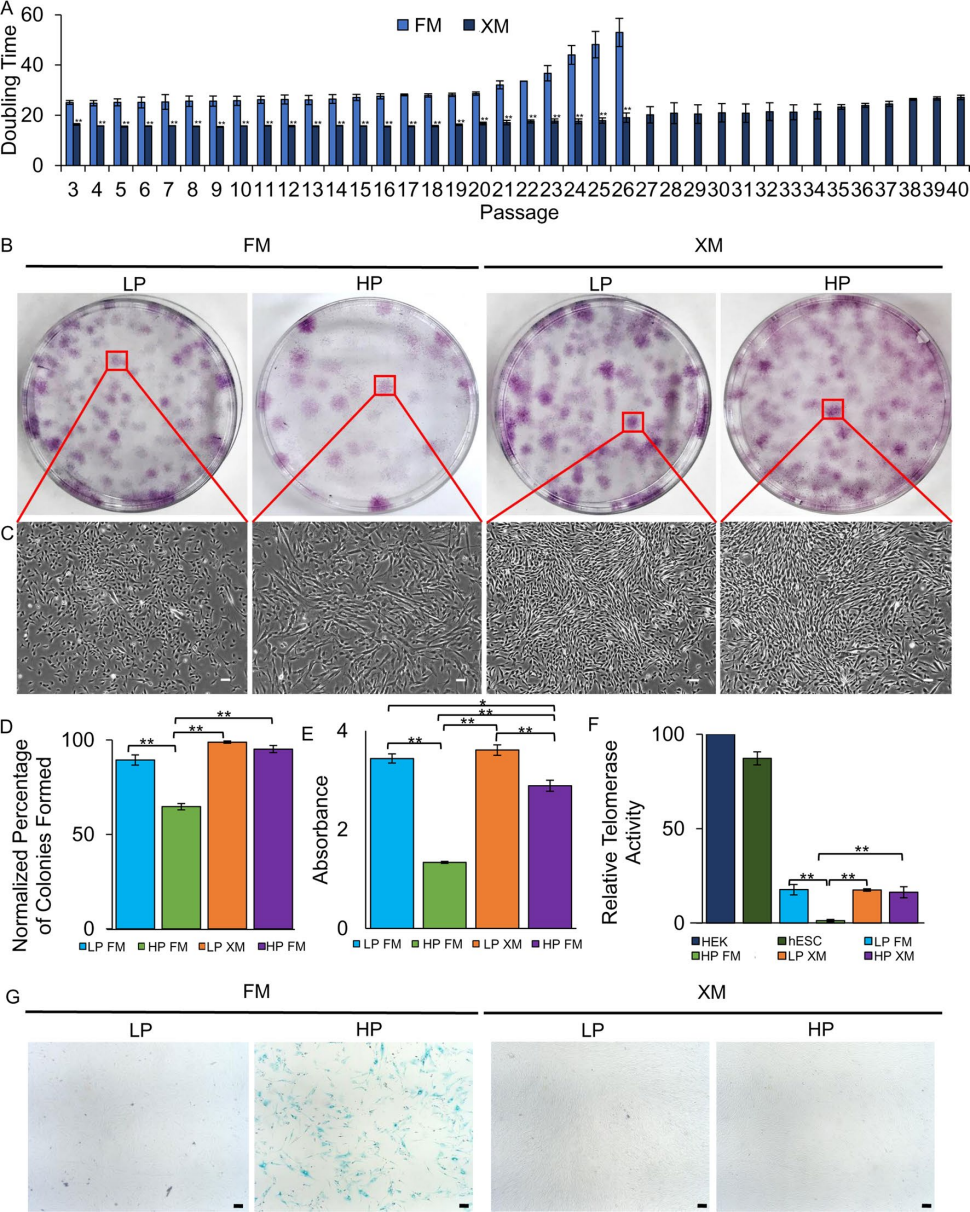
Proliferation and colony-forming efficiency assays of LP and HP pMSCs grown in FM and XM. **a** Doubling time of pMSCs **b**, **c** Crystal violet stained colonies of pMSCs and phase contrast showing the morphology of a single colony of pMSCs performed in triplicate. Scale bars represent 100 μm (magnification: 4×). **d** Percentage of colony formation of LP and HP pMSCs performed in triplicate. **E** Proliferation of pMSCs as determined using BrdU proliferation kit performed in triplicate. **f** Relative telomerase activity in various cell types as determined using the qRT-PCR-based TRAP assay performed in triplicate. All values are reported as telomerase activity relative to HEK cells. Growth of pMSCs in FM yielded cells with significantly reduced doubling time and CFE, less compact colonies, and decreased proliferation rate and relative telomerase activity. **g** Light microscopy images of SA-β-gal stained pMSCs. Blue staining indicates senescent cells. Scale bars represent 100 μm (magnification: 4×). Any results showing **p ≤ 0.01 and *p ≤ 0.05 were deemed statistically significant

XM also maintained a low doubling time (DT) even up to P40, but the DT was gradually and rapidly increased in FM (Fig. 2a). The cell practically stopped growing soon after P25. Similar to the MSC-markers and DT, the clonogenicity of pMSCs was maintained at a high level, 99% and 95% CFE in LP and HP pMSCs, respectively, in XM, but it was significantly reduced, 89% and 65% CFE in LP and HP pMSCs, respectively FM (Fig. 2b–d). Furthermore, cell proliferation assay demonstrated higher BrdU uptake (OD, 3.6 and 2.9) in LP and HP pMSCs, respectively, grown in XM compared to LP and HP pMSCs (OD, 3.4 and 1.3, respectively) grown in FM (Fig. 2e). In addition, the low-level telomerase activity was maintained to near LP pMSCs (17.5%) in HP cells (16.4%) grown in XM. Still, it was a significant decrease (1.3%) in HP pMSCs in FM relative to the telomerase activity in HEK cells (100%) and human ESCs (87.2%) (Fig. 2f). Analysis of SA-β-gal in pMSCs revealed that HP but not the LP cells cultured in FM expressed the SA-β-gal (Fig. 2g). No expression of SA-β-gal was observed in both LP and HP MSCs grown in XM. Taken together, the rate of proliferation, CFE, and expression of CD90 and CD49f were significantly reduced in pMSCs upon passaging in FM. However, these characteristics were well maintained in XM-grown cells, indicating the gene expression does not significantly change between LP and HP, when grown in XM.

### Trilineage differentiation potential of pMSCs

**Fig. 3:**
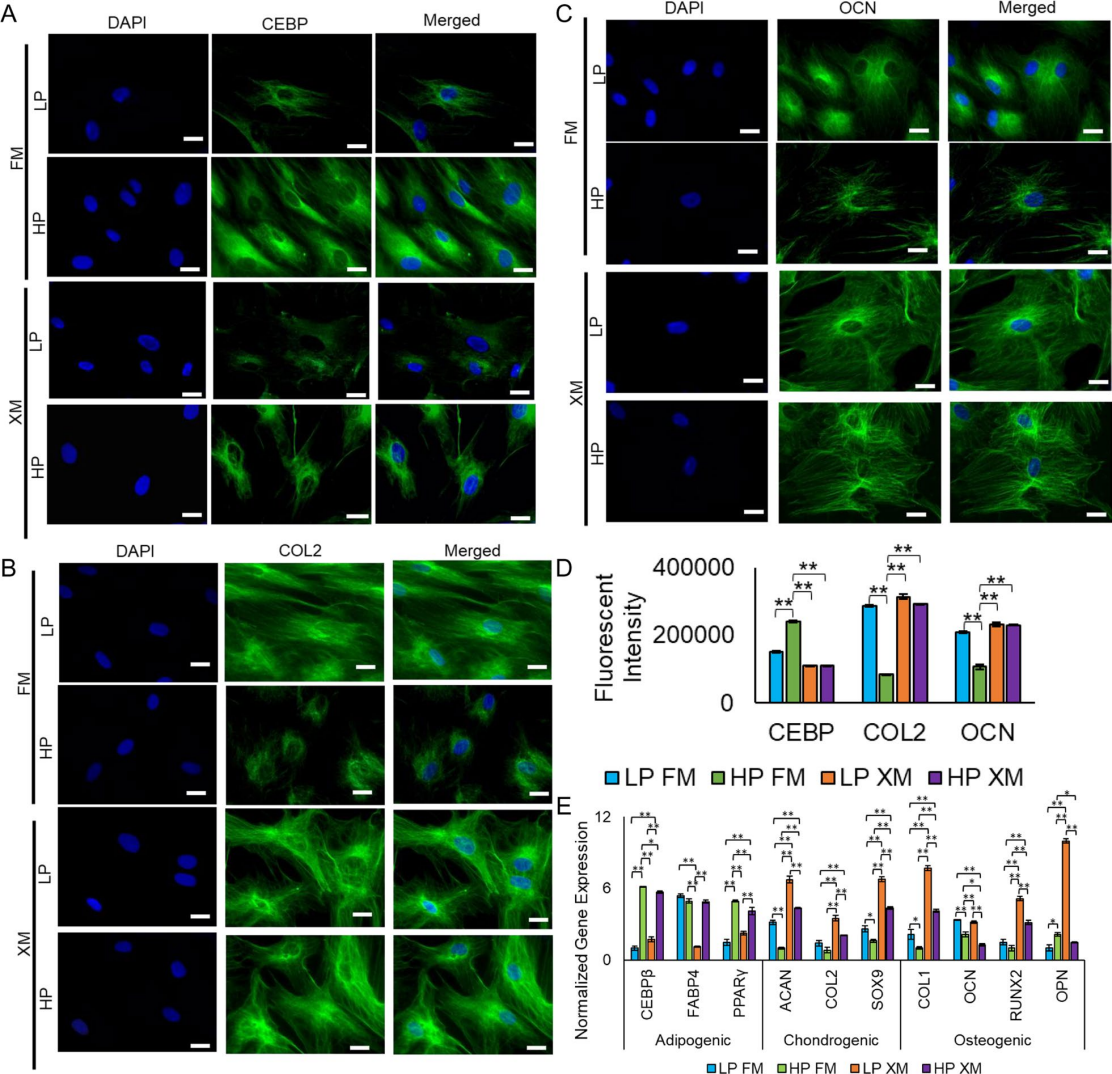
Determination of differentiation potential of LP and HP pMSCs grown in FM and XM. **a**–**c** Fluorescence microscopic images and fluorescence intensity of the derivatives stained with CEBP, COL2, and OCN antibodies representing chondrogenic, osteogenic, and adipogenic lineages, respectively. **d** Quantification of fluorescent intensity of immunostained derivatives of pMSCs performed in triplicate. Scale bars represent 400 μm (magnification: 40×). **e** Expression of adipogenic (CEBPβ, FABP4, and PPARγ), chondrogenic (SOX9, ACAN, and COL2), and osteogenic (COL1, RUNX2, OPN, and OCN) genes in the differentiated derivatives. Gene expression was normalized to GAPDH and ACTIN, and error bars represent the standard deviations of the triplicate measurements. HP pMSCs grown in FM showed a greater propensity to differentiate toward adipogenic lineage. Any results showing **p ≤ 0.01 and *p ≤ 0.05 were deemed statistically significant

LP and HP pMSCs grown in FM and XM differentiated into chondrogenic, osteogenic, and adipogenic lineages based on the staining of cells with toluidine blue, alizarin red, and oil red O [4, 5]. The differentiated derivatives expressed adipogenic, chondrogenic, and osteogenic proteins, CEBP, COL2, and OCN, respectively, as determined by immunostaining (Fig. 3a–c). Interestingly, adipogenic derivatives of HP pMSCs cultured in FM showed a significant increase in the expression of CEBP compared to the cells grown in XM, whereas chondrogenic and osteogenic derivatives of LP and HP pMSCs cultured in XM and LP pMSCs cultured in FM had a significant higher expression of COL2 and OCN, respectively, compared to the derivatives of the cells grown in in FM (Fig. 3d). Overall, pMSCs cultured in the XM showed a greater tendency to differentiate toward the chondrogenic and osteogenic lineage, whereas HP pMSCs cultured in the FM showed a greater tendency to differentiate toward the adipogenic lineage. qRT-PCR analysis of the differentiated derivatives of HP pMSCs cultured in FM had an upregulation of adipogenic genes (*CEPβ, FABP4, and PPARγ*), while those of LP pMSCs cultured in XM had higher expression for chondrogenic (*ACAN, COL2, and SOX9*) and osteogenic (*COL1, OCN, RUNX2, and OPN*) genes (Fig. 3e).

### Differential gene expression

Based on the analysis of cell morphology, DT, CFE, telomerase activity, and differentiation of LP and HP pMSCs cultured in FM and XM, we envisioned changes in the gene expression. Therefore, we first analyzed the overall gene expression in pMSCs grown in two different media. This analysis showed distinct differences in cells grown in FM and XM (Additional file 3: Fig. S3a). Evidently, the cells grown in XM had similar gene expression with slight differences between LP and HP pMSCs. Likewise, cells grown in FM had similar gene expression with slight differences between LP and HP pMSCs. However, there were significant differences between the cells grown in FM and XM.

Next, we compared the differentially expressed genes (DEGs) between the various groups using no plots (Additional file 3: Fig. S3b). The number of DEGs between the pMSCs grown in FM and XM was significantly more compared to the cells grown in the same medium, irrespective of the passage. The least DEGs were observed between the LP and HP cells grown in XM.

Venn diagram also revealed that most DEGs (749) were found between HP cells grown in FM vs LP cells grown in XM followed by 403 DEGS in LP cells grown in FM vs HP cells grown in XM. In other groups, the difference in DEGs was less than 200. Only 21 DEGs between LP and HP cells were grown in XM (Additional file 3: Fig. S3c). This suggests that pMSCs grown XM were more similar compared to cells grown in FM.

**Fig. 4:**
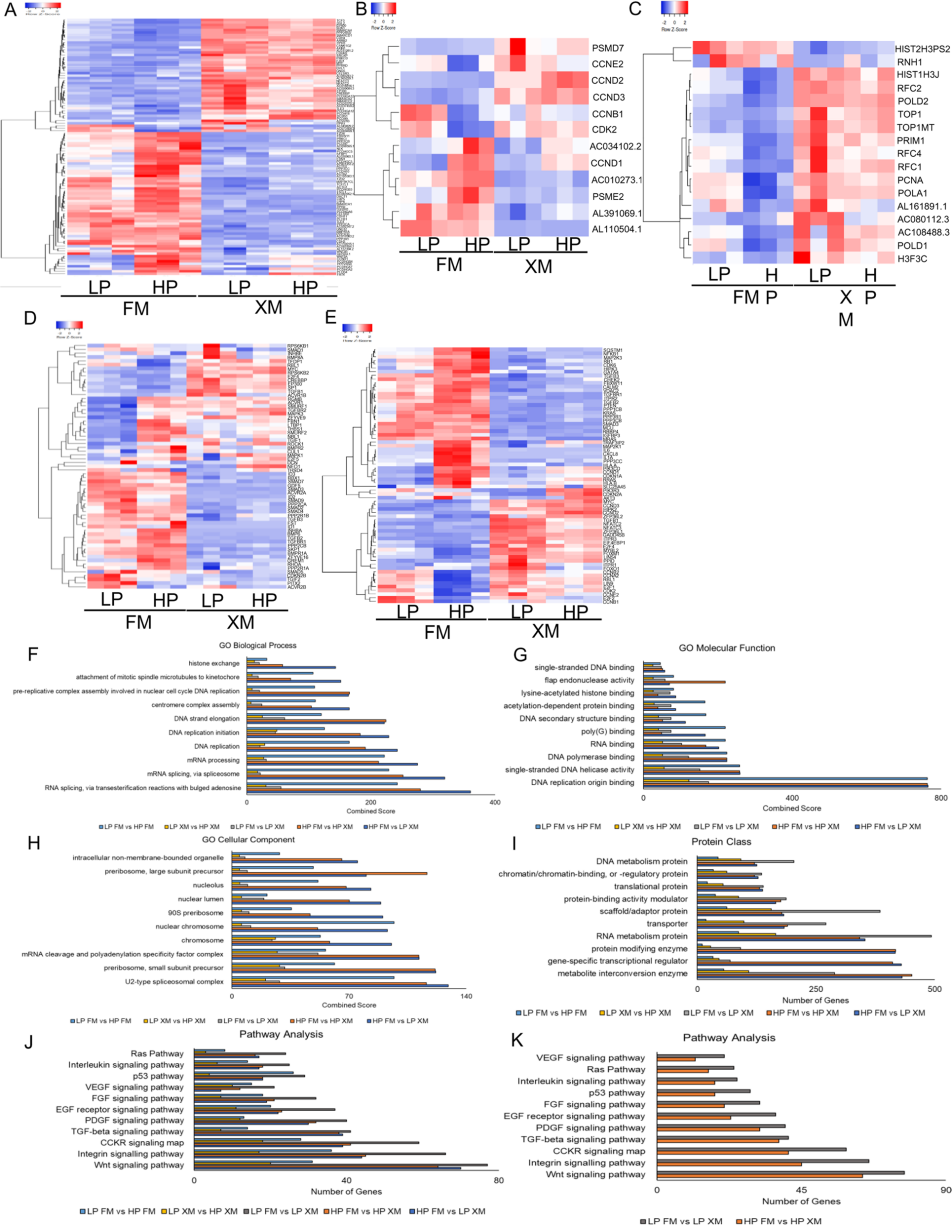
Transcriptional analysis in LP and HP pMSCs grown in FM and XM. **a**–**e** RNA-seq results show the Wnt signaling, cell cycle, DNA replication, TGF beta, and senescence genes, respectively, performed in triplicate. Up- and downregulated genes are red and blue, respectively. HP pMSCs grown in FM displayed downregulation of Wnt signaling, cell cycle, and DNA replication genes but upregulation of TGF beta and senescence genes. **f**–**h** Enrichment of upregulated DEGs associated with the biological processes, molecular function, and cellular compartment, respectively, was performed with Benjamini–Hochberg FDR at *p* < 0.05 using DESeq2. **i**–**j** Upregulated DEGs associated with protein classes and signaling pathways, respectively, were determined by PANTHER analysis. **k** Enrichment of pathway genes when DEGs were compared between HP XM vs HP FM and LP XM vs LP FM. The Enrichr analysis generated the combined score (*p*-value multiplied by the z-score)

Heat map data in Fig. 4a–e show the DEGs related to Wnt signaling, cell cycle, DNA replication, TGFβ, and senescence pathways in pMSCs. The results revealed that many Wnt signaling pathway genes were upregulated in pMSCs grown in XM but downregulated in FM (Fig. 4a, Additional file 3: Fig. S3d and Additional file 4: Fig. S4a–b).

The prominent upregulated Wnt pathway genes that may positively affect cell proliferation included *DVL2, CSNK1G2, AXIN1, SRCAP, PRKCD, FZD1, MYC, DVL1, CREBBP*, and *TLE3*. In addition, several genes of SNF/SWI family (*SMARCA4, SMARCD1, SMARCA2,* and *SMARCC2*) involved in chromatin modeling and two genes (*EP300* and *EP400*) associated with histone acetylation were upregulated in XM cells. In contrast, several Wnt genes (*PPP2CB, SFRP1, TCF7L1, TLE4, PPP2CA, CCND1, PPP2R5B*, and *FZD2*) that may be negatively affecting cell proliferation was upregulated in cells grown in FM but downregulated in XM. In addition, two genes (*TGFβR1* and *BMPR1A*) and one gene (*SMARCA1*) associated with differentiation and chromatin remodeling, respectively, were also upregulated in cells grown in FM. Importantly, the expression of these genes was more significant in HP than LP pMSCs, suggesting that FM-grown cells had reduced self-renewal capacity or undergoing differentiation.

Furthermore, LP and HP pMSCs cultured in XM showed upregulation of cell cycle genes (*PSMD7, CCNE2, CCND2*, and *CDK2*) related to proliferation. In LP and HP pMSCs cultured in FM, several lncRNAs (*AL110504.1, AL391069.1*, and *AC010273*.1) were upregulated (Fig. 4b, Additional file 3: Fig. S3e, and Additional file 4: Fig. S4c–d). It would be interesting to determine the function of these lncRNAs. HP pMSCs cultured in FM had only two upregulated genes (*HIST2H3PS2* and *RNH1*) associated with the DNA replication, while pMSCs cultured in XM had several upregulated genes (*RFC2, POLD2, RFC4, PCNA,* and *RBL1*) related to the DNA replication (Fig. 4c, Additional file 3: Fig. S3f, and Additional file 4: Fig. S4e–f).

pMSCs grown in FM and XM also had DEGs associated with TGFβ signaling (Fig. 4d, Additional file 3: Fig. S3g, and Additional File 4: Fig. S4g–h). Several of DEGs (*TFDP1, RBL1, MYC, RPS6KB2, E2F4*, and *SP1*) and two DEGs (*CREBBP* and *EP300*) were associated with cell cycle and chromatin remodeling, respectively. These DEGs were upregulated in pMSCs grown in XM but downregulated in FM, whereas five DEGs (*ID3, RBX1, PPP2CA, PPP2R1B*, and *PPP2CB*) involved in the negative regulation of the cell cycle were upregulated in pMSCs grown in FM. Not surprisingly, several DEGs (*SMAD7, GDF5, SMAD3, ACVR2A, SMAD2, SMAD4, TGFβ3, INHBA, BMP6, TGFβ2, TGFβR1, BMPR1A*, and *ZFYVE16*) associated with TGFβ signaling were upregulated in pMSCs grown in FM but not in XM. Additionally, pMSCs grown in the FM had more DEGs *(CXCL8, IL6, IL1A, CDKN2A*, and *CDKN1A*) involved in senescence compared to cells grown in the XM (Fig. 4e, Additional file 3: Fig. S3h, and Additional file 4: Fig. S4i–j). On the other hand, DEGs related to self-renewal, such as *E2F1, CDK2, MYC, FOXO1*, and *CCND2* were upregulated in cells grown in XM.

Furthermore, analysis of the DEGs, *p*-value < 0.05 using gene ontology (GO) revealed that they are associated with biological processes, molecular function, and cellular component ontologies. The most significant enrichment of biological processes such as histone exchange, attachment of mitotic spindle microtubules to kinetochore, pre-replicative complex assembly involved in nuclear cell cycle DNA replication, centromere complex assembly, DNA strand elongation, DNA replication initiation, DNA replication, mRNA processing, mRNA splicing via spliceosome, and RNA splicing via transesterification reactions with bulged adenosine as nucleophile was revealed in LP pMSCs grown in XM vs HP cells grown in FM when compared with all the groups (Fig. 4f). In contrast, a significant variation in the enrichment of molecular functions, such as single-stranded DNA binding, flap endonuclease activity, lysine-acetylated histone binding, acetylation-dependent protein binding, DNA secondary structure binding, poly(G) binding, RNA binding, DNA polymerase binding, single-stranded DNA helicase activity, and DNA replication origin binding (Fig. 4g) and cellular components such as intracellular non-membrane-bound organelle, preribosome large subunit precursor, nucleolus, nuclear lumen, 90S preribosome, nuclear chromosome, chromosome, mRNA cleavage and polyadenylation specificity factor complex, preribosome small subunit precursor, and U2-type spliceosomal complex (Fig. 4h) was observed in the cells grown in FM vs XM irrespective of the cell passage. These observations suggest that differences between molecular functions and cellular component processes could be due to the culture media.

We then subjected the RNA-seq data to PANTHER analysis, and the results are shown in Fig. 4i–k. Evidently, most proteins classes (DNA metabolism protein, chromatin/chromatin binding or regulatory protein, translational protein, protein-binding activity modulator, scaffold/adaptor protein, transporter, and RNA metabolism protein, except protein modifying enzyme, gene-specific transcriptional regulator, and metabolite interconversion enzyme) (Fig. 4i) and signaling pathways (DNA replication, Ras, Interleukin signaling, TGFβ signaling, Cadherin signaling, p53, integrin signaling, PDGF signaling, CCKR signaling map, and Wnt signaling pathways (Fig. 4j) were enriched in LP cells grown in XM vs LP cells grown in FM when compared with all the groups. Pathway analysis comparing LP FM vs LP XM and HP FM vs HP XM revealed more enrichment in pathways such as Wnt, TGFβ, PDGF, and VEGF in the LP FM vs LP XM group (Fig. 4k).

### Interactome analysis

Interactome analysis using GOnet showed upregulation of selected DEGs associated with cell cycle and DNA replication positive interaction between the LP and HP cells grown in XM but not in FM (Additional file 5: Fig. S5a, b). The selected upregulated DEGs associated with proliferation showed a positive interaction among the genes expressed in LP and HP cells grown in XM but not FM (Additional file 5: Fig. S5c, d). In contrast, selected DEGs associated with senescence revealed positive interaction among the genes expressed in LP and HP cells grown in FM but not in XM (Additional file 5: Fig. S5e, f).

### Analysis of nucleosome occupancy

**Fig. 5:**
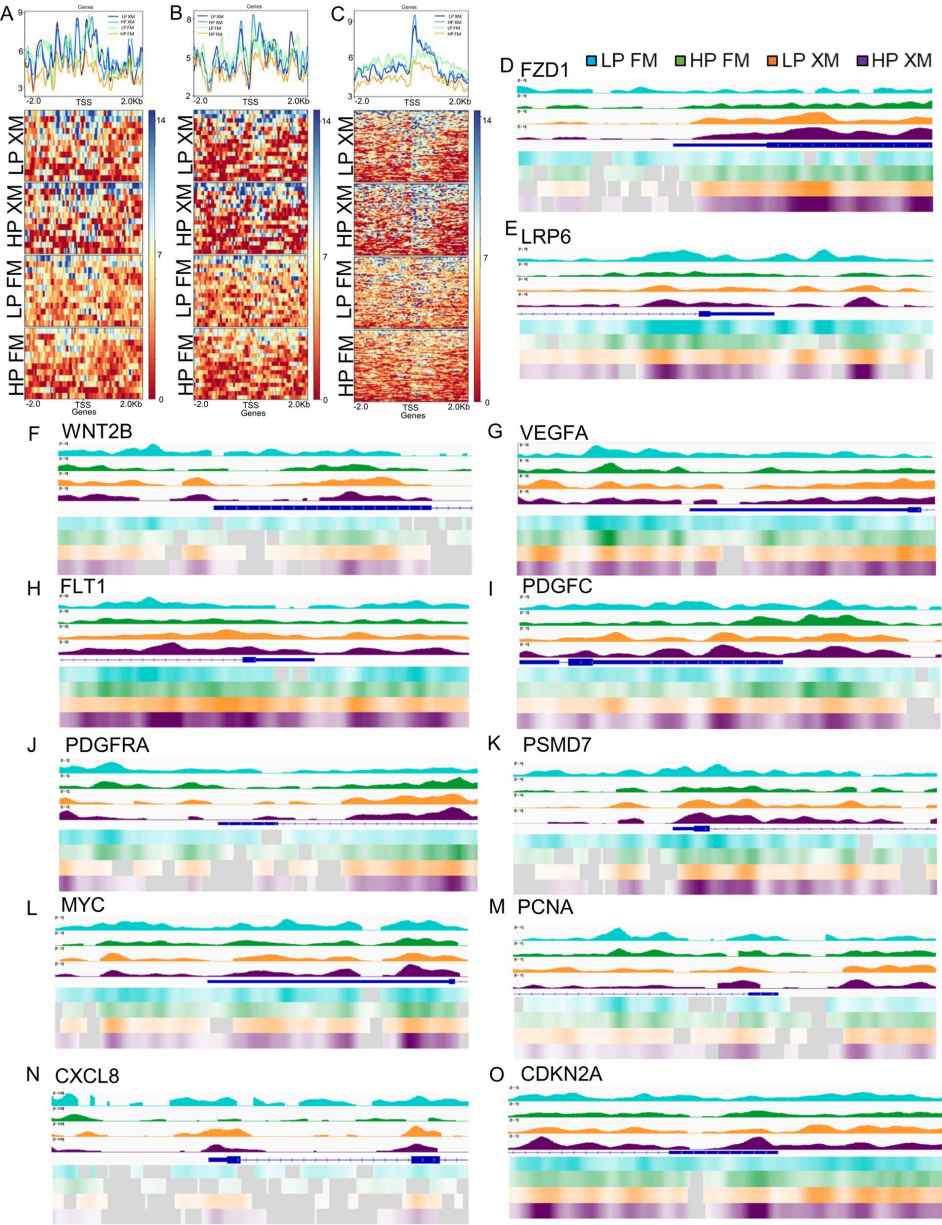
Nucleosome mapping and genome browser shots showing nucleosome occupancy determined by Mnase-seq and IGV analysis **a**–**c** Nucleosome mapping showing cell cycle, DNA replication, and senescence genes, respectively. **d**–**f** Wnt pathway genes (FZD1, LRP6, and Wnt2B), **g**–**j** VEGF/PDGF pathway genes (VEGFA, FLT1, PDGFC, and PDGFRA), **k** cell cycle gene (PSMD7), **L**–**M** Self-renewal and DNA replication genes (MYC and PCNA), and **n**–**o** senescence (CXCL8 and CDKN2A). Data showed less nucleosome occupancies in pMSCs cultured in XM compared to FM in cell cycle and DNA replication genes. In contrast, more nucleosome occupancies in pMSCs cultured in XM than FM were found in senescence genes. IGV mapping showed that FZD1, LRP6, Wnt2B, VEGFA, FLT1, PDGFC, PDGFRA, PSMD7, MYC, and PCNA genes had less nucleosome occupancies around the promoters, suggesting the higher expression in pMSCs grown in XM than FM, whereas CXCL8 and CDKN2A had higher nucleosome occupancies around the promoters, suggesting their lower expression in pMSCs grown in XM compared to FM. LP and HP pMSCs grown in FM (blue and green, respectively), LP and HP pMSCs grown in XM (orange and purple, respectively)

In addition, we performed the MNase-seq analysis of pMSCs cultured in the two different media (Fig. 5a–c). These results showed more nucleosome occupancy in LP and HP pMSCs cultured in the FM compared to LP pMSCs grown in the XM in genes involved in some cellular functions, particularly cell cycle and DNA replication (Fig. 5a, b respectively). On the other hand, less nucleosome occupancy was observed in HP pMSCs cultured in the FM compared to the cells grown XM in genes involved with the senescence pathway (Fig. 5c). This suggests that genes related to cell cycle and DNA replication in pMSCs cultured in XM, and genes related to senescence have lower histone occupancies that could promote higher expression in HP pMSCs cultured in FM.

### Integrative genomics viewer (IGV) mapping

Since RNA-seq analysis revealed passaging and medium composition affected the expression, we examined MNase-seq data to evaluate nucleosome occupancies of selected genes involved in self-renewal, cell cycle, DNA replication, differentiation, and senescence. IGV maps show less nucleosome occupancies in the area around the promoters of genes associated with Wnt (*FZD1, LRP6, and Wnt2B*), VEGF/PDGF (*VEGFA, FLT1, PDGFC,* and *PDGFRA*), cell cycle (PSMD7), self-renewal (MYC), and DNA replication (PCNA) (Fig. 5d–m, respectively) in pMSCs cultured in XM compared to FM. In contrast, senescence genes (CXCL8 and CDKN2A) had less nucleosome occupancy in pMSCs cultured in FM than XM (Fig. 5n–o). These results suggest that upregulation of the self-renewal and proliferation genes show lower nucleosome occupancies in cells grown in XM consistent with their higher expression. In contrast, senescence genes are induced in cells grown in FM, consistent with their higher expression.

### Expression of selected genes

**Fig. 6:**
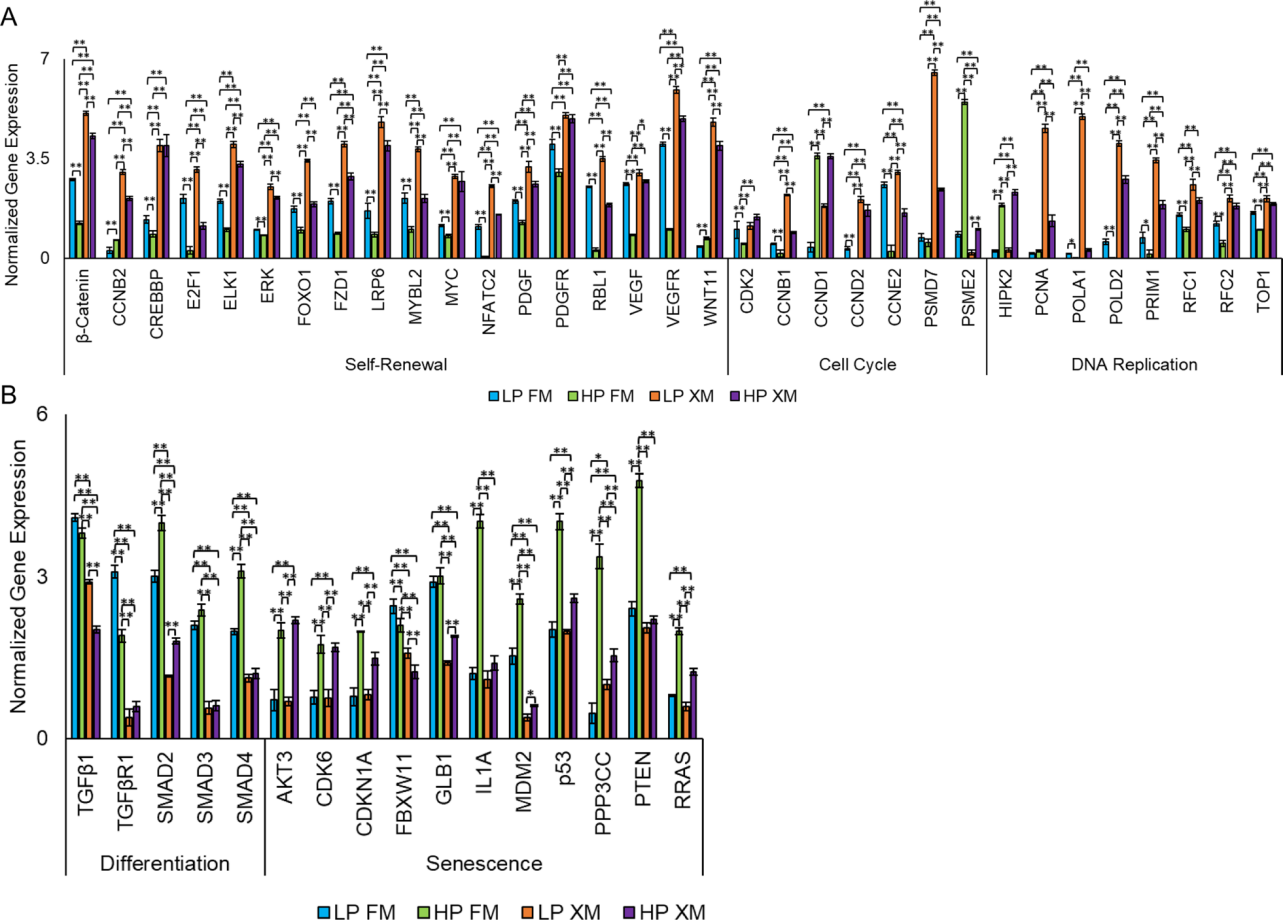
Relative expression of genes in pMSCs cultured in FM and XM. **a** Genes involved in self-renewal, cell cycle, and DNA replication. **b** Genes involved in differentiation and senescence. Gene expression was normalized to GAPDH and ACTIN, and error bars represent the standard deviations of the triplicate measurements. Any results showing **p ≤ 0.01 and *p ≤ 0.05 were deemed statistically significant

We validated the expression of selected DEGs by qRT-PCR. The results depicted in Fig. 6a, show self-renewal genes (*β-catenin, CCNB2, CREBBP, E2F1, ELK1, ERK, FOXO1, FZD1, LRP6, MYBL2, NFATC2, PDGF, PDGFR, RBL1, VEGF, VEGFR*, and *Wnt11*) upregulated in pMSCs cultured in XM. In addition, several cell cycle genes (*CCNB1, CCND2, CCNE2*, and *PSMD7*) were upregulated in LP pMSCs grown in XM. Only one cell cycle gene was upregulated in HP pMSCs cultured in FM and XM (*CCND1*), and only *PSME2* was upregulated in HP cells grown in FM. Several genes related to DNA replication (*PCNA, POLA1, POLD2, PRIM1, RFC1, RFC2,* and *TOP1*) were upregulated in pMSCs grown in XM compared to FM. Only one DNA replication gene (*HIPK2*) was upregulated in HP pMSCs grown in FM, but its significance remains to be investigated. In contrast, several differentiation genes (*TGFβ1, TGFβR1, SMAD2, SMAD3*, and *SMAD4)* were upregulated in pMSCs cultured in FM (Fig. 6b) than XM. Furthermore, we found several senescence genes (*CDKN1A, GLB1, IL1A, MDM2, p53, PPP3CC, PTEN*, and *RRAS*) highly upregulated in HP pMSCs cultured in FM. These results suggest XM supported self-renewal and FM induced differentiation and senescence in pMSCs.

### Effects of Wnt and TGFβ agonist and antagonist on pMSCs

**Fig. 7:**
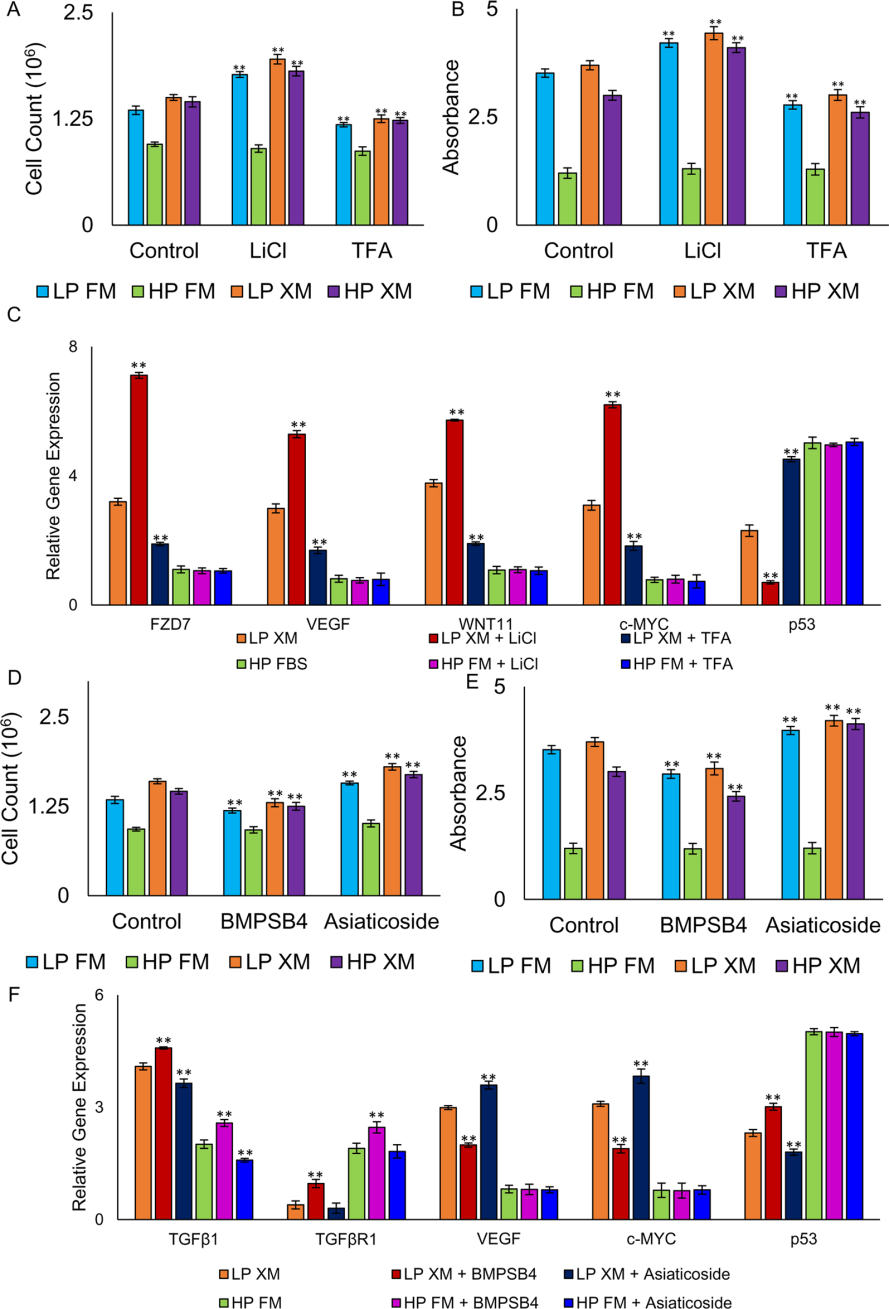
Effect of Wnt and TGFβ agonist and antagonist on pMSCs cultured in FM and XM. **a**, **b** The proliferation of pMSCs cultured in the 10 mM Wnt agonist, LiCl, and 500 nM Wnt antagonist, TFA, determined by direct counts and using BrdU proliferation assay. **c** Relative expression of selected genes involved in self-renewal in pMSCs cultured in media containing LiCl or TFA compared to the control. **d**, **e** The proliferation of cells cultured in 0.25 mM TGFβ agonist, BMPSB4, and 0.125 mM TGFβ antagonist, Asiaticoside, determined by direct counts and using BrdU proliferation assay. **f** Relative expression of selected genes involved in TGFβ and self-renewal in cells supplemented with BMPSB4 and Asiaticoside compared to the control Gene expression was normalized to GAPDH and ACTIN, and error bars represent the standard deviations of the triplicate measurements. Significance was measured in comparison of the control. Any results showing **p ≤ 0.01 and *p ≤ 0.05 were deemed statistically significant

Since our transcriptional analysis indicates that Wnt signaling and TGFβ signaling influence self-renewal, we investigated the effects of agonist and antagonist of Wnt and TGFβ on pMSCs. The results depicted in Fig. 7a, b revealed a significant increase in cell counts and BrdU uptake in LP and HP pMSCs grown in XM and LP pMSCs grown in FM but not in HP cells grown in FM treated with the Wnt agonist LiCl. In contrast, cells treated with Wnt antagonist, TFA had significantly lower cell counts and BrdU uptake, except in HP cells grown in FM. Gene expression analysis of LP cells grown in XM and treated with LiCl had a significant increase in the selected self-renewal genes, FZD7, VEGF, Wnt11, and c-MYC, but they were downregulated when these cells were treated with TFA (Fig. 7c). However, the expression of p53 was significantly decreased and increased in these cells treated with LiCl and TFA, respectively. Similar results were found in HP cells grown in XM and LP cells grown in FM. LP and HP pMSCs cultured in XM and LP pMSCs cultured in FM treated with the TGFβ agonist, BMPSB4, revealed a significant decrease in cell counts and BrdU uptake compared to the control (Fig. 7d–e). In contrast, cells grown in XM and LP cells grown in FM treated with TGFβ antagonist Asiaticoside showed significant increase in cell counts and BrdU uptake compared to control cells. Transcriptional analysis of LP pMSCs cultured in XM treated with BMPSB4 showed a significant increase in the expression of TGFβ1, TGFβR1, and p53 but a significant decrease in VEGF and c-MYC compared to the control (Fig. 7f). HP cells grown in FM treated with BMPSB4 only revealed a significant increase in TGFβ1 and TGFβR1 expression. In contrast, LP cells grown in XM treated with Asiaticoside demonstrated a significant increase in VEGF and c-MYC but a significant decrease in TGFβ1 and p53. Asiaticoside treatment significantly reduced TGFβ1 levels in HP cells grown in FM, XM, and LP cells grown in FM. These results confirm that passaging of cells in FM induces loss of self-renewal with concomitated downregulation of Wnt signaling and upregulation of TGFβ signaling.

## Discussion

There are different sources from which MSCs can be obtained [1–3], but their properties can vary in terms of self-renewal, differentiation, and their ability to reduce inflammation and regulate the immune system [3, 4, 38, 39]. Furthermore, their properties can change during the process of propagation [17–21, 25] since MSCs tend to lose their capacity for self-renewal or proliferation with each passage [26, 27]. This poses a significant challenge to achieving consistent, reliable results in clinical applications.

This study investigated the changes in pMSCs grown in FM and XM. We first observed the cell morphology and surface markers of pMSCs. The cells grown in FM exhibited an elongated and larger cell structure compared to those grown in XM and reduced CD90 and ITGA/CD49f expression. Previous studies have demonstrated that CD90 levels decrease during MSC differentiation [40, 41], while CD49f is a self-renewal marker expressed in over 30 types of stem cells [42, 43].

Upon further examination of pMSCs in FM, it was found that their doubling time rapidly increased over time. In contrast, cells grown in XM had a steady doubling time that slowly increased, consistent with previous studies [22–24]. Additionally, the CFE of pMSCs was significantly lower in FM than in XM. The highest CFE and more compact colonies were seen in LP cells grown in XM. The BrdU assay further revealed reduced proliferation of pMSCs grown in FM. Adult MSCs typically have relatively low or no telomerase activity, a self-renewal marker, that has been shown to decrease as stem cells differentiate or enter into senescence [44–48]. pMSCs revealed lower telomerase activity than hESCs and HEK cells. While relative telomerase activity remained almost the same in XM, it was lost in FM when grown to HP. HP pMSCs grown in FM also expressed SA-β-gal, which validates the decrease in self-renewal properties found in the results. Increased SA-β-gal activity in senescent cells due to the increased expression of GLB1 [49]. A prior study found that MSCs at a late stage of passage, based on increased doubling time, had an increased expression of SA-β-gal [50]. This aligns with our results, which showed that as pMSCs grown in FM passaged, their doubling time exponentially increased, leading to their expression of SA-β-gal. However, pMSCs grown in XM were able to undergo multiple passages without a significant increase in their doubling time and without the expression of SA-β-gal.

We investigated whether the media used affects the ability of pMSCs to differentiate into different cell types. Both XF and FM allowed pMSCs to differentiate into adipogenic, chondrogenic, and osteogenic lineages. However, HP pMSCs in FM had a higher tendency to differentiate toward the adipogenic lineage, while pMSCs grown in XM and LP cells grown in FM showed a greater tendency to differentiate toward the chondrogenic and osteogenic lineages. While MSCs can lose their ability to self-renew and differentiate into three different cell types, they can still differentiate into the adipogenic lineage [51–54].

Several studies have revealed a decrease in the self-renewal and differentiation potential of MSCs, but the molecular changes responsible for this shift have not been thoroughly investigated. Our transcriptomic and MNase-seq analyses of pMSCs showed significant differences in gene expression between pMSCs grown in the two different conditions. Volcano plot analysis of DEGs in pMSCs also revealed more significant differences when comparing the two media suggesting that the medium composition has a greater impact on the fate of pMSCs.

Wnt signaling is crucial for self-renewal and differentiation in various stem cells, including MSCs [55, 56]. In XM-grown pMSCs, the expression of Wnt genes related to positive regulation of self-renewal was increased, as expected. However, pMSCs cultured in FM had upregulated Wnt genes related to negative regulation of self-renewal or induction of differentiation. Furthermore, XM-grown cells also had increased expression of two Notch signaling genes, SRCAP and TLE3, which are believed to regulate the self-renewal capacity and multipotency of stem cells [57, 58]. In contrast, pMSCs cultured in FM had upregulated expression of genes that promote differentiation [59, 60]. Additionally, SMARCA1/SNF2L, a chromatin remodeling ATPase involved in DNA damage response [61], was upregulated in cells grown in FM. Several DEGs of the cadherin superfamily were found in pMSCs cultured in both FM and XM, which play a role in cell-to-cell adhesion. These findings suggest that XM but not FM supports the maintenance of self-renewal in pMSCs.

The regulation of cell proliferation is dependent on the interaction between Cyclin/CDK and Myc/E2F [62–65]. During the G1 phase of the cell cycle, CDK4 complexes with CCND, leading to the phosphorylation of RB. This results in the upregulation of transcription for CDK2 together with CCNE, which allows the cells to advance into the S phase [63, 66]. MYC also plays a role in facilitating E2F promotion, which helps cells transition from G1 to S phase. Our research found that E2F1 and CDK2 were upregulated in cells grown in XM, while LP cells cultured in FM showed the same increase. In contrast, several proliferation genes were upregulated in pMSCs cultured in XM, and downregulated in pMSCs in FM. Interestingly, cells grown in FM showed upregulation of several lncRNA that could be epigenetic regulators of transcriptional and translational levels [67]. Further studies are needed to understand the induction and role of these lncRNA in determining the fate of pMSCs.

The TGFβ pathway genes related to self-renewal were upregulated in pMSCs grown in XM but downregulated in those grown in FM [68]. Additionally, CREBBP and EP300, which regulate gene expression through histone acetylation [69, 70], were also upregulated in cells grown in XM. Several genes of the TGFβ pathway that induce differentiation [60] were upregulated in FM-grown pMSCs. Furthermore, three genes (PPP2CA, PPP2R1B, and PPP2CB) of the TGFβ pathway that negatively regulate the cell cycle by dephosphorylating RB [71, 72] were upregulated in FM. These findings align with the involvement of the TGFβ signaling pathway in regulating various cellular processes, including proliferation and differentiation [29]. As FM-grown cells had progressively increased DT and reduced CFE, and downregulated self-renewal genes, we wondered if these changes further impacted the fate of pMSCs toward senescence.

The process of aging and cancer development is strongly influenced by senescence, which involves the accumulation of cell damage and dysfunction over time. When cells become senescent, they stop growing in the G1 phase to prevent DNA replication in damaged cells [73, 74], and in the G2 phase to block mitosis in the presence of DNA damage [75]. MSCs undergo senescence as they are passaged [76–80]. It was found that HP pMSCs grown in FM had the highest number of senescence genes, while DEGs in LP and HP pMSCs grown in XM were mainly inhibitory to senescence. In XM, pMSCs showed upregulated expression of genes related to positive self-renewal regulation. Conversely, DEGs in FM-grown pMSCs were related to TGFβ signaling pathway as discussed above. HP pMSCs grown in FM had higher expression of the senescence marker, GLB1 (SA-β gal), which is consistent with the higher SA-β-gal stain expression observed in HP cells grown in FM. These findings were consistent with a previous report on adipose-derived MSCs showing an increase in SA-B gal-positive cells upon passaging [81].

The CHEK2 gene was found to be more active in pMSCs that were grown in FM. This gene activates CDKN1A (p21) and inhibits CDK-cyclin complexes like CDK2, which could have led to the cell cycle arrest in HP pMSCs grown in FM [82, 83]. Comparing the DEGs in pMSCs grown in different media, there were significant differences in biological processes, molecular function, and cellular components. However, there was less variation between LP and HP pMSCs. Pathway analysis showed that Wnt, TGFβ, PDGF, and VEGF pathways were more enriched in LP FM vs LP XM group. GOnet analysis showed that certain cell cycle, DNA replication, and proliferation genes interact positively in pMSCs grown in XM. Conversely, selected senescence genes had positive interaction among the genes expressed in pMSCs grown in FM.

We conducted MNase-seq analysis to study the results at a chromatin level. Our findings revealed that in pMSCs cultured in the FM, there was more nucleosome occupancy in DNA replication genes and cell cycle compared to the XM. Conversely, we observed less nucleosome occupancy in senescence genes in HP pMSCs cultured in the FM but not in the XM. Our research also demonstrated that cell cycle and DNA replication genes were upregulated in XM-grown cells, while senescence genes were upregulated in FM-grown cells. The lower nucleosome occupancies in these genes corresponded with upregulation. We found that the area surrounding promoters of genes related to Wnt and VEGF/PDGF signaling pathways had low nucleosome occupancy. Additionally, genes associated with cell cycle, self-renewal, and DNA replication also showed low nucleosome occupancy in XM-grown pMSCs. On the other hand, selected senescence DEGs in FM-grown pMSCs had less nucleosome occupancy, which is consistent with their higher expression, which was confirmed through qRT-PCR.

We delved deeper into the role of Wnt signaling in self-renewal by studying the impact of LiCl (an agonist) and Fz7-21 TFA (an antagonist) on pMSCs' cell proliferation rate. LiCl affects Wnt signaling by phosphorylating Ser9, which inhibits GSK3 and stabilizes β-catenin [84–86], and also lowers p53 [84]. Conversely, Wnt antagonists such as DKK1 have been shown to increase p53 expression [87]. We therefore examined the effect of LiCl and TFA on self-renewal genes and found that LiCl upregulates them while TFA decreases their expression, further supporting the involvement of the Wnt pathway in pMSC self-renewal. Our findings also revealed that BMPSB4 (a TGFβ agonist) promotes senescence of pMSCs, while Asiaticoside (a TGFβ antagonist) inhibits it. TGFβ agonist supplementation led to a decrease in cell count and BrdU uptake and reduced expression of self-renewal genes. Previous studies have shown that TGFβ1 treatment in bone marrow-derived MSCs increases SA-β-gal and mitochondrial reactive oxygen production compared to the control [33], while a TGFβ inhibitor promotes cell proliferation and prevents senescence in MSCs [88]. Our results and past studies further confirmed our observation of higher TGFβ pathway expression in HP cells grown in FM based on RNA-seq and reduced self-renewal and increased SA-β-gal expression in our findings. Therefore, it is crucial to investigate these pathways further in future studies to optimize MSC maintenance and regenerative properties.

**Fig. 8:**
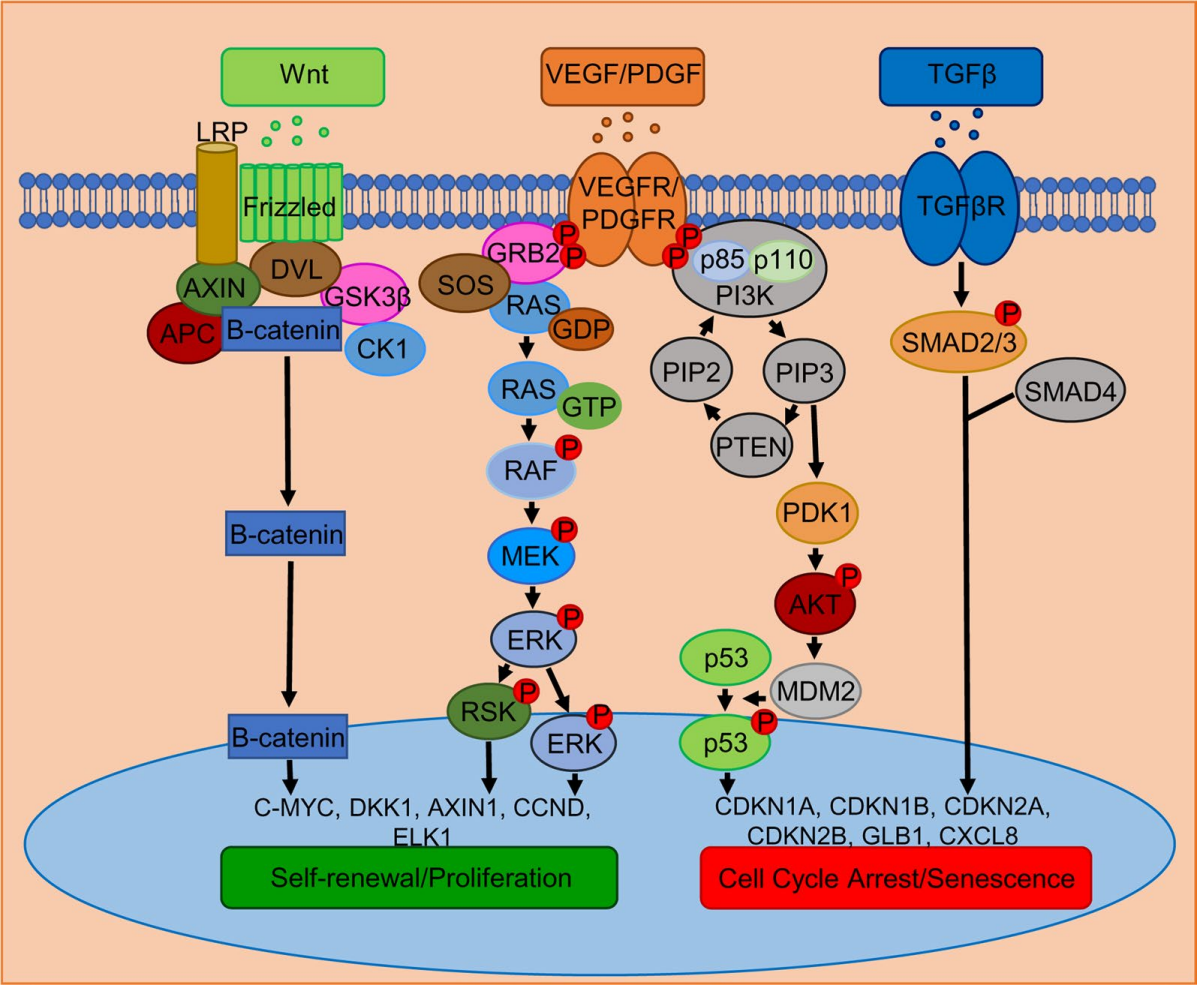
Proposed Wnt, VEGF/PDGF, and TGF signaling pathways mediating self-renewal and cell cycle arrest in pMSCs. In XM, the Wnt protein binds to the Frizzled receptor, keeping the Wnt pathway active. This destabilizes the destruction complex (DVL/AXIN/APC/GSK3β/CK1) and allows β-catenin to promote the expression of self-renewal genes in the nucleus. Additionally, growth factors like VEGF and PDGF play a role in pMSC proliferation in XM by phosphorylating their respective receptors. This recruits the adaptor protein GRB2 and the nucleotide exchange factor SOS, which activate RAS, RAF, MEK, and ERK in sequence. Phosphorylated ERK enters the nucleus and activates the transcription of cell proliferation genes such as c-MYC. Alternatively, ERK activates RSK, which then activates proliferation genes. In FM, PI3K is activated, leading to an increase in PTEN and PDK1 expression, which activates AKT. Activated AKT presumably phosphorylates MDM2, modulating p53 activity responsible for senescence gene expression. Senescence is gradually induced by TGFβ interaction with TGFβR, which phosphorylates SMAD2/3, moving to the nucleus with SMAD4 to turn on genes involved in cell cycle arrest

Our proposed mechanism for self-renewal or cell arrest is illustrated in Fig. 8, based on the collected evidence.

## Conclusion

In conclusion, we found that pMSCs in FM undergo changes in morphology and trilineage differentiation capacity, as well as a decrease in proliferation and CFE, and an increase in SA-β-gal expression. Based on transcriptomic analysis, it appears that Wnt signaling is involved in maintaining the self-renewal and proliferation of pMSCs, while TGFβ signaling is responsible for gradual loss of self-renewal and initiation of senescence. Our experiments with Wnt and TGFβ agonists and antagonists support the conclusion that Wnt signaling regulates self-renewal in pMSCs, while TGFβ regulates senescence. These findings have implications for the expansion of pMSCs in large-scale clinical studies aimed at treating degenerative diseases.

### Supplementary Information

**Additional file 1**. List of primer sequences used in qRT-PCR.

**Additional file 2**. Graphical percentage of surface markers in LP and HP pMSCs grown in FM and XM. The results were performed in triplicate. Any results showing **p ≤ 0.01 and *p ≤ 0.05 were deemed statistically significant.

**Additional file 3**. Plotting and Venn diagrams of DEGs in LP and HP pMSCs cultured in two different media. (**a**) Expression heat map of sample-to-sample distances on the matrix of variance-stabilized data for overall gene expression. Darker blue colors indicate a similar correlation (the color key is in arbitrary units). Clustering indicates that pMSCs cultured in XM were similar. Likewise, pMSCs cultured in FM were similar but different from cells grown in XM. (**b**) Volcano plots showing raw z-scores of RNA-seq log2 transformed values of the DEGs. (**c**) A significant number of DEGs in cells grew in FM and XM. (**d**–**h**) DEGs related to Wnt, cell cycle, DNA replication, TGF beta, and senescence, respectively, at a cutoff of *p* < 0.05.

**Additional file 4**. Comparative analysis of different groups of genes in LP and HP pMSCs grown in FM and XM. (**a**–**b**): Wnt signaling genes in LP FM vs LP XM and HP FM vs HP XM, respectively. (**c**–**d**): Cell cycle genes in LP FM vs LP XM and HP FM vs HP XM, respectively. (**e**–**f**): DNA replication genes on LP FM vs LP XM and HP FM vs HP XM, respectively. (**g**–**h**) TGF beta genes on LP FM vs LP XM and HP FM vs HP XM, respectively. (**i**–**j**) Senescence genes on LP FM vs LP XM and HP FM vs HP XM, respectively.

**Additional file 5**. Interactome mapping of the selected cell cycle, DNA replication, senescence, and proliferation genes on pMSCs. (**a**–**b**): Interaction of upregulated genes involved in cell cycle and DNA replication, respectively, in LP and HP pMSCs cultured in XM. (**c**–**d**): Interaction of upregulated genes involved in cell proliferation in LP and HP pMSCs, respectively, cultured in XM. (**e**–**f**): Interaction of upregulated senescence genes in LP and HP pMSCs, respectively, cultured in FM.

## Data Availability

The raw and processed sequencing data generated in this study have been deposited in the NCBI GenBank database, accession code PRJNA928451. All data needed to evaluate the conclusions are present in the paper and/or the Supplementary Materials.

## Abbreviations

BMPSB4: BMP signaling agonist sb4
C: Control
CFE: Colony-forming efficiency
CD: Cluster of differentiated
DEGs: Differentially expressed genes
DT: Doubling time
FM: Fetal bovine serum medium
HEK: Human embryonic kidney cells
hESCs: Human embryonic stem cells
HP: High passage
HP FM: HP pMSCs grown in FM
HP XM: HP pMSCs grown in XM
LiCl: Lithium chloride
LP: Low passage
LP FM: LP pMSCs grown in FM
LP XM: LP pMSCs grown in XM
MNase-seq: Micrococcal nuclease sequencing
MSCs: Mesenchymal stem cells
P: Passage
p: Primitive
qRT-PCR: Quantitative reverse transcriptase-polymerase chain reaction
RNA-seq: RNA-sequencing
SA-β-gal: Senescence-associated β-galactosidase
SEM: Standard error of the mean
XM: Xeno-free medium
