# Supplementary figures and images for: Regulation of self-renewal and senescence in primitive mesenchymal stem cells by Wnt and TGFβ signaling
Source: Stem Cell Res Ther. 2023 Oct 26;14:305. doi: 10.1186/s13287-023-03533-y (PMC10601332; doi:10.1186/s13287-023-03533-y)

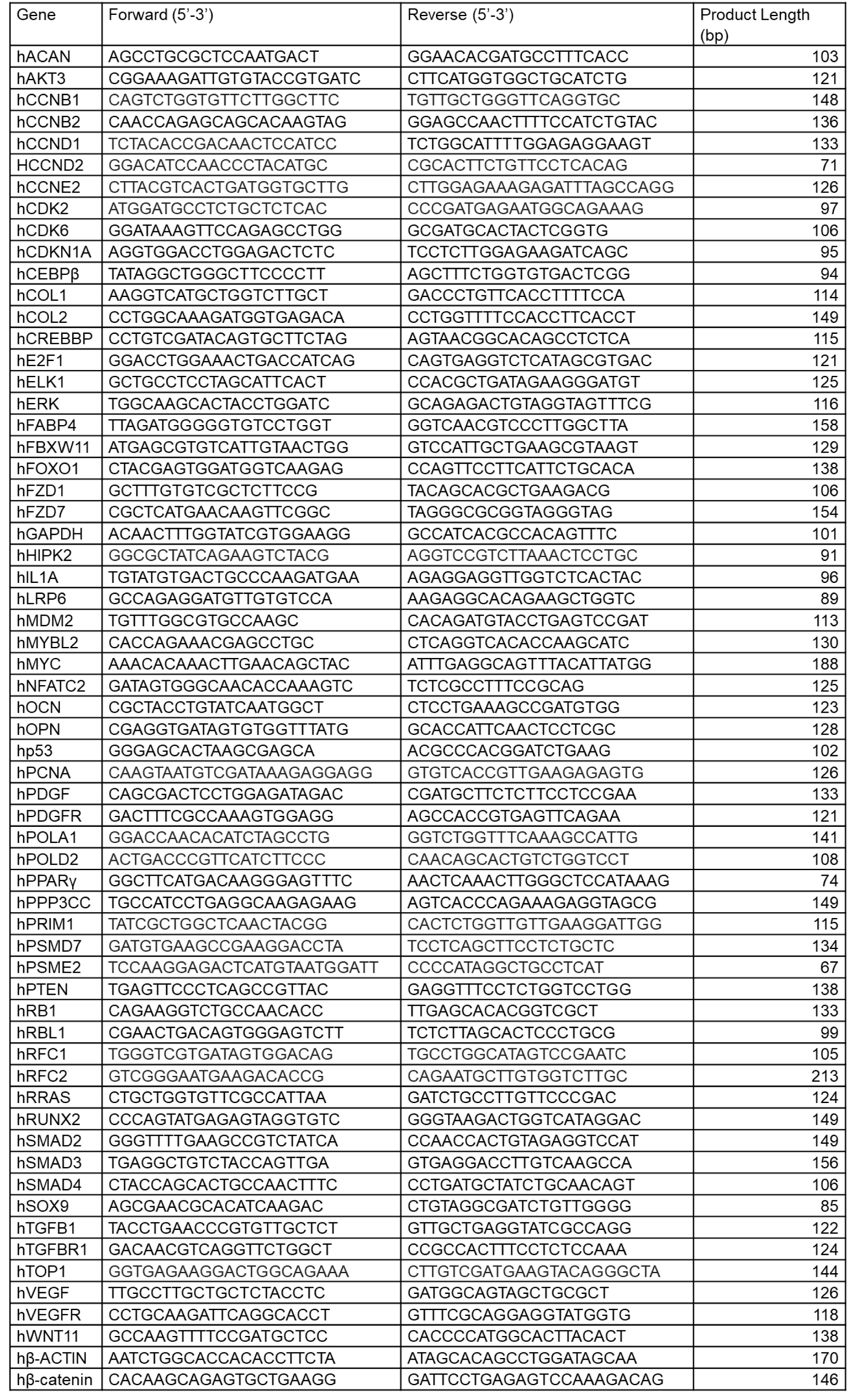

Supplement: Supplementary file 1 — Additional file 1. List of primer sequences used in qRT-PCR. [file 13287_2023_3533_MOESM1_ESM.tif]

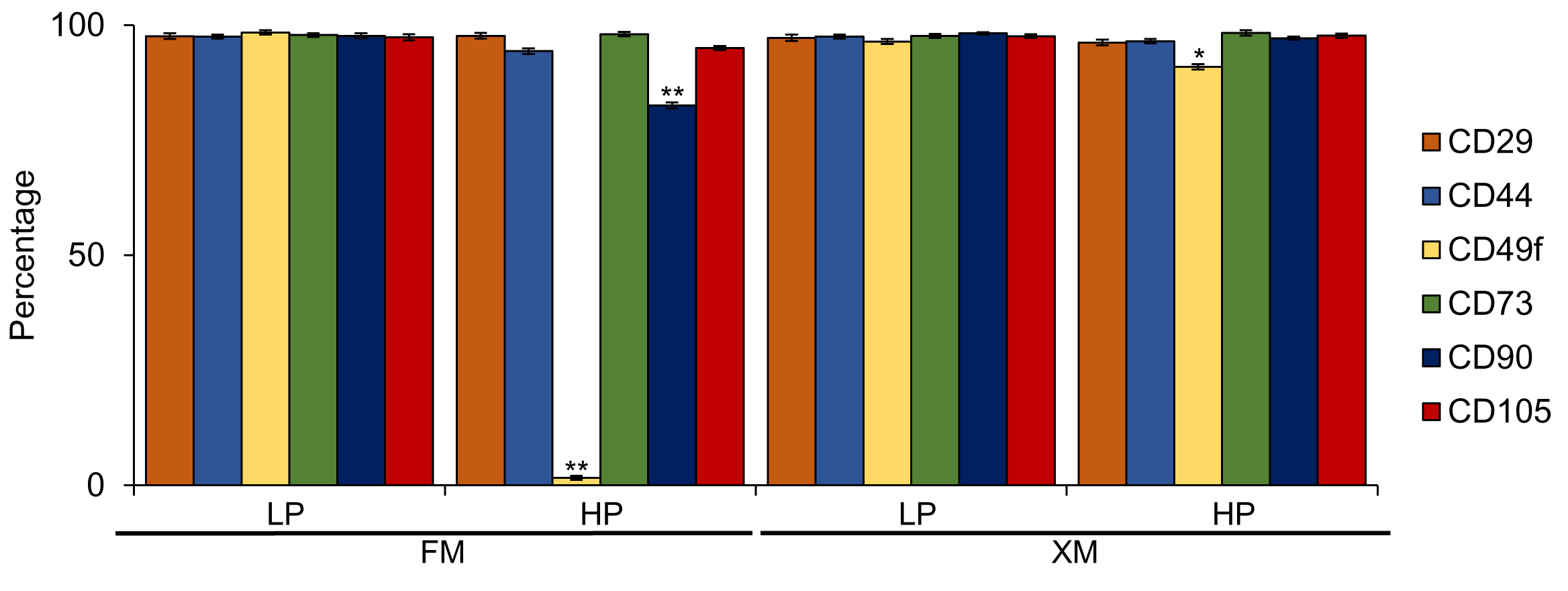

Supplement: Supplementary file 2 — Additional file 2. Graphical percentage of surface markers in LP and HP pMSCs grown in FM and XM. The results were performed in triplicate. Any results showing **p ≤ 0.01 and *p ≤ 0.05 were deemed statistically significant. [file 13287_2023_3533_MOESM2_ESM.tif]

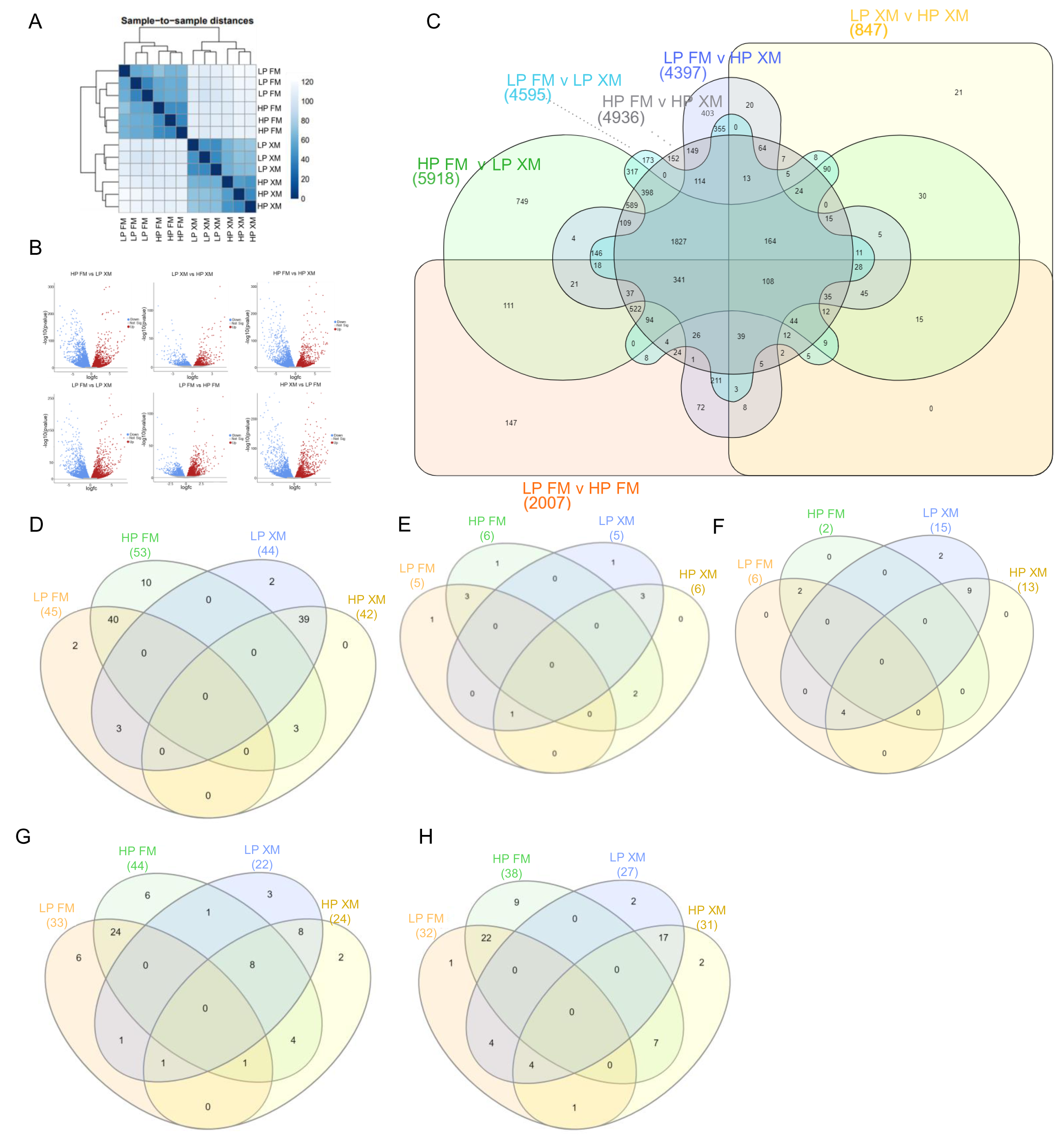

Supplement: Supplementary file 3 — Additional file 3. Plotting and Venn diagrams of DEGs in LP and HP pMSCs cultured in two different media. (a) Expression heat map of sample-to-sample distances on the matrix of variance-stabilized data for overall gene expression. Darker blue colors indicate a similar correlation (the color key is in arbitrary units). Clustering indicates that pMSCs cultured in XM were similar. Likewise, pMSCs cultured in FM were similar but different from cells grown in XM. (b) Volcano plots showing raw z-scores of RNA-seq log2 transformed values of the DEGs. (c) A significant number of DEGs in cells grew in FM and XM. (d–h) DEGs related to Wnt, cell cycle, DNA replication, TGF beta, and senescence, respectively, at a cutoff of p < 0.05. [file 13287_2023_3533_MOESM3_ESM.tif]

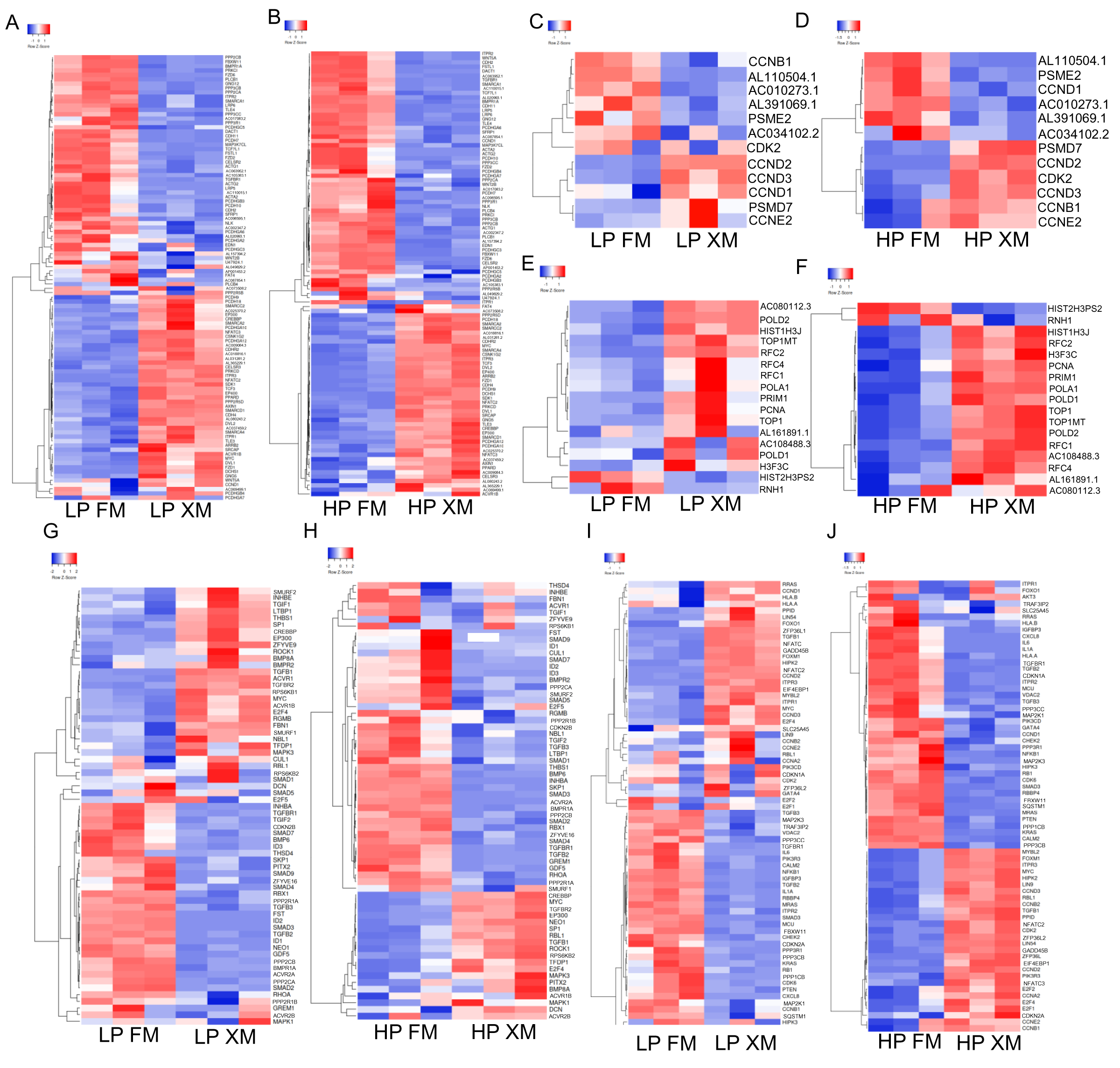

Supplement: Supplementary file 4 — Additional file 4. Comparative analysis of different groups of genes in LP and HP pMSCs grown in FM and XM. (a–b): Wnt signaling genes in LP FM vs LP XM and HP FM vs HP XM, respectively. (c–d): Cell cycle genes in LP FM vs LP XM and HP FM vs HP XM, respectively. (e–f): DNA replication genes on LP FM vs LP XM and HP FM vs HP XM, respectively. (g–h) TGF beta genes on LP FM vs LP XM and HP FM vs HP XM, respectively. (i–j) Senescence genes on LP FM vs LP XM and HP FM vs HP XM, respectively. [file 13287_2023_3533_MOESM4_ESM.tif]

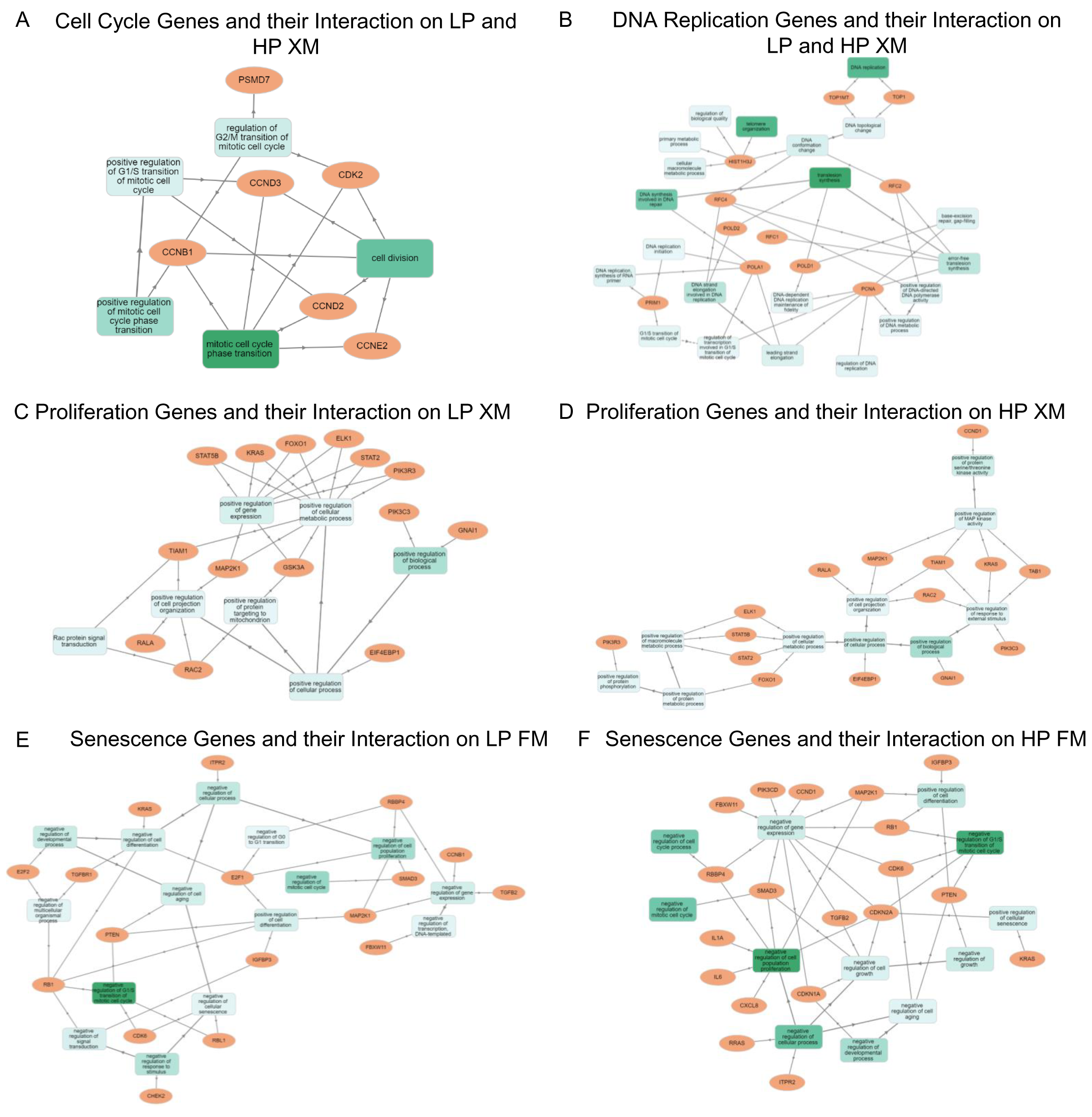

Supplement: Supplementary file 5 — Additional file 5. Interactome mapping of the selected cell cycle, DNA replication, senescence, and proliferation genes on pMSCs. (a–b): Interaction of upregulated genes involved in cell cycle and DNA replication, respectively, in LP and HP pMSCs cultured in XM. (c–d): Interaction of upregulated genes involved in cell proliferation in LP and HP pMSCs, respectively, cultured in XM. (e–f): Interaction of upregulated senescence genes in LP and HP pMSCs, respectively, cultured in FM. [file 13287_2023_3533_MOESM5_ESM.tif]
